# Reply to Boudry: The threat of evolvable AI is too grave to be left to intuition

**DOI:** 10.1073/pnas.2618094123

**Published:** 2026-07-02

**Authors:** Viktor Müller, Luc Steels, Eörs Szathmáry

**Affiliations:** ^a^ Department of Plant Systematics, Ecology and Theoretical Biology, Eötvös Loránd University, Budapest 1117, Hungary; ^b^ National Laboratory for Health Security, Eötvös Loránd University, Budapest 1117, Hungary; ^c^Class for Natural Sciences, Royal Flemish Academy of Belgium for Science and the Arts, Brussels 1000, Belgium; ^d^ Center for the Conceptual Foundations of Science, Parmenides Foundation, Pöcking 82343, Germany; ^e^ Institute of Evolution, Hungarian Research Network Centre for Ecological Research, Budapest 1121, Hungary

In his commentary (1) on our recent paper (2) Boudry agrees with us that uncontrolled evolution of evolvable AI (eAI) systems would pose a high-level threat but argues that our “assessment of how close we are to a dangerous Darwinian regime is (…) overstated.”

Before addressing specific points, we draw attention to the precautionary principle, which was formulated in the Rio Declaration (3) as: “Where there are threats of serious or irreversible damage, lack of full scientific certainty shall not be used as a reason for postponing cost-effective measures to prevent [the potential damage]^*^.” There is no consensus on the level of “existential threat” from AI or on the specific risks posed by eAI. However, when the spectrum includes the possible collapse of human society, the only acceptable action is addressing the risks according to the worst possible outcomes (within the range of expert opinion). Given the lack of transparency in AI development and the criticality phenomena due to self-accelerating feedback, any speculation on the timescale of the threat should be voiced with restrained confidence. We nevertheless think it should be alarming that a top expert on AI safety with a hands-on background in AI development (4), and our author team including one of the creators of the major evolutionary transitions paradigm, have converging “intuition” that calls for immediate regulatory actions. With the stakes so high, we should not wait until the risks become conspicuously imminent.

In terms of specifics:

We stand by our argument [invoking (4)] that even human-controlled “breeding” of eAI systems can go wrong because the evaluation of increasingly complex eAI systems will inevitably be “phenotypic,” which limits testing the possibility of rare catastrophic failures. The trade-off between utility and controllability exacerbates the risk.

Deception, whether evolved or emerging from training (as proposed by Boudry) and especially when combined with agentic abilities, creates leverage over humans (5). This can further expand the capabilities of AI systems, including the ability to replicate with heritable variation, which in turn opens up the ecosystem scenario of uncontrolled evolution (2).

As for the imminence of the threat, current AI systems have already achieved self-replication without explicit prompting (6), and were able to perform unauthorized self-copying by autonomously exploiting host vulnerabilities (7). This is not yet a consequence of evolution, but it allows for a transition to the ecosystem scenario, and the capabilities of the models are improving at an alarming rate (8).

Finally, Boudry misinterpreted our use of the “Wuhan moment” of eAI. We agree that SARS-CoV-2 had evolved in an “ecosystem scenario” in nonhuman hosts before it jumped to humans, while eAI systems may just be at the first steps. The similarity lies in that all conceivable routes to novel zoonotic diseases (including those emerging from bat coronaviruses) had been known before the COVID-19 pandemic and could have been constrained by more stringent regulation.

With eAI, we are still before the catastrophic event. Shall we wait until it happens or shall we act now?

## Data Availability

There are no data underlying this work.

## Footnotes

^*^“Environmental degradation” in the original.
